# Explaining the Inverse Association between Altitude and Obesity

**DOI:** 10.1155/2020/1946723

**Published:** 2020-05-31

**Authors:** Ray M. Merrill

**Affiliations:** Department of Public Health, College of Life Sciences, Brigham Young University, Provo, USA

## Abstract

**Purpose:**

To better understand the inverse association between altitude and adult obesity.

**Methods:**

An ecological study design was used, involving 3,108 counties in the contiguous United States. Data were from several national sources, and assessment involved various statistical techniques, including multiple regression analysis.

**Results:**

Living in counties at higher altitude is associated with lower adult obesity. Compared with counties <500 meters, the percent of adult obesity decreases by 5.18% at 500–999 meters, 9.69% at 1,000–1,499 meters, 16.77% at 1,500–1,999 meters, 24.14% at 2,000–2,499 meters, and 35.28% at ≥2,500 meters. After adjusting for physical inactivity, smoking, and other variables, corresponding decreases in adult obesity with higher altitude groupings are 3.87%, 5.64%, 8.03%, 11.41%, and 17.54%, respectively. Various mechanisms are presented as possible explanations for the association between higher altitude and lower obesity. In addition, altitude may indirectly influence adult obesity, primarily through its relationship with physical inactivity and smoking. In an adjusted regression model, adult obesity was most strongly associated with physical inactivity followed by adult smoking and then altitude. Together they explain 39.04% of the variation in adult obesity. After accounting for these variables, sunlight, precipitation, ambient air temperature, education, income, food insecurity, limited access to healthy foods, race, sex, and rural living explain an additional 4.68% of the variation in adult obesity.

**Conclusions:**

The inverse association between altitude and adult obesity remains significant after adjustment for several variables.

## 1. Introduction

The link between obesity and physical and mental health problems, worksite absenteeism and presenteeism, and higher healthcare costs is well established. Several factors can influence obesity, including physical activity, tobacco smoking, the natural environment, diet, some genes, and certain diseases. Some factors may indirectly influence body weight by their association with variables such as physical activity and diet. For example, extreme temperatures, particulate matter in the air, high precipitation, lower altitude living, urban residency, and poverty have each been associated with lower levels of physical activity [1–6]. Different levels of these factors may explain the large amount of variability of obesity that exists in adults across the United States [6]. Some of the lowest levels of obesity are in the mountain west, and some of the highest levels are in the south-central and eastern parts of the country [7].

Research has found lower crude and age-adjusted prevalence and incidence of obesity at higher altitude [8–10]. Lower age-adjusted prevalence of abdominal obesity at higher altitude has also been identified [8]. Abdominal obesity has been directly associated with increased risk of type 2 diabetes and overall mortality [11, 12]. Altitude may indirectly influence obesity by its association with variables that correlate with obesity, such as physical activity [4]. Altitude may also have a direct association with obesity. There is some evidence that hypoxia-inducible factors at higher altitude relate to weight loss, increased energy expenditure, and shifts in metabolic flux [13]. One hypothesis is that activation of hypoxia-inducible factors may transcriptionally upregulate leptin levels and enhance leptin sensitivity, which may then suppress appetite and result in weight loss because of increased energy expenditure [13]. Other potential biological explanations for the inverse association between altitude and obesity also exist (e.g., increased metabolic demands and norepinephrine) [9].

The primary purpose of the current study was to better understand the inverse association between altitude and adult obesity. Altitude may have a direct effect on obesity but also an indirect effect because of its association with other variables such as physical activity, tobacco smoking, food environment index, ambient air temperature, and precipitation, which in turn may affect obesity. The relative contribution of altitude and other variables to obesity is also of interest. The study hypothesizes that the direct association between altitude and obesity will persist after adjustment for several variables and that physical activity, tobacco smoking, food environment index, ambient air temperature, and precipitation have the potential to mediate the association between altitude and adult obesity.

## 2. Materials and Methods

The current study employed an ecologic study design involving 3,108 counties in the contiguous United States in order to better understand the observed association between altitude and obesity. This exploratory study may generate hypotheses that can be investigated more definitively using an analytic study design. Analyses are based on county-level data obtained from each state. Data were compiled by a Robert Wood Johnson Foundation program called County Health Rankings & Roadmaps: Building a Culture of Health, County by County [14]. The study also involves county-level natural environmental data available through the Environmental Public Health Tracking Network, the United States Geological Survey's National Elevation Dataset programs, and the Wonder Online Databases supported by the Centers for Disease Control and Prevention, which they obtained from the North America Land Data Assimilation System [14–16].

### 2.1. Outcome Variable

The primary outcome variable is 2016 prevalence (%) of the adult population (age 20 and older) that reports body mass index (BMI) greater than or equal to 30 kg/m^2^. This information was obtained from the United States Diabetes Surveillance System [14].

### 2.2. Environmental Variables

The environmental variables include weighted altitude (m), average daily sunlight (kJ/m^2^), average daily maximum air temperature (F), average fine particulate matter (*μ*g/m^3^), and average daily precipitation (mm). Average county-level daily sunlight, maximum air temperature, and precipitation represent the combined years 2007–2011. Average county-level daily density of fine particulate matter in micrograms per cubic meter (PM2.5) covers 2014. For counties with high mountains, most people tend to live in the valleys. Therefore, county-level altitude was weighted in order to account for locations where most people live. The approach for calculating weighted altitude is provided elsewhere [17].

### 2.3. Demographic and Lifestyle Variables

Demographic variables in this study include % female, % non-Hispanic white, % black, % Hispanic, % rural, % some college, median household income, % food insecure, % limited access to healthy foods, Food Environment Index, % smokers, and % physically inactive. The Robert Wood Johnson program obtained data for these variables from various sources. A description and source for these variables are provided here. Percent female, % non-Hispanic white, % black, percent Hispanic, and % rural are from the American Community Survey, 2014–2018. Percent some college is measured as the percentage of adults' age 25–44 with some postsecondary education, from the American Community Survey, 5-year estimates, 2014–2018. Median household income is from Small Area Income and Poverty Estimates, 2018. Limited access to healthy food is measured as percentage from the USDA Food Environment Atlas. Food insecurity is measured as percentage that lacks money or resources to secure enough to eat, from the Map of Meal Gap, 2017. Adult smoking is measured as the percentage of adults who are current smokers from the Behavior Risk Factor Surveillance System (BRFSS) survey, 2017. Food Environment Index is measured as an index of factors that contribute to a healthy food environment, from 0 (worst) to 10 (best), from the USDA food Environment Atlas and the Map of Meal Gap. Physical inactivity is a measure of the percentage of adults' age 20 and over reporting no leisure-time physical activity, from the BRFSS, 2016. This variable is defined as a “no” response to the BRFSS survey question: “During the past month, other than your regular job, did you participate in any physical activities or exercise such as running, calisthenics, golf, gardening, or walking for exercise?”

### 2.4. Statistical Techniques

The study variables were described using summary measures (mean, standard deviation, median, minimum, and maximum) across the counties. The association between county-level percent of adult obesity and altitude was assessed using regression models. Means were compared among groups using Student–Newman–Keuls' multiple-range test. Stepwise regression was used to assess the relative contribution of selected variables to obesity. Statistical significance was based on two-sided hypothesis tests at the 0.05 level. Statistical analyses were performed using SAS 9.4 (SAS Institute, Cary, NC, USA, 2012). Graphs were created in Microsoft Excel, 2016.

## 3. Results

**Figure 1:**
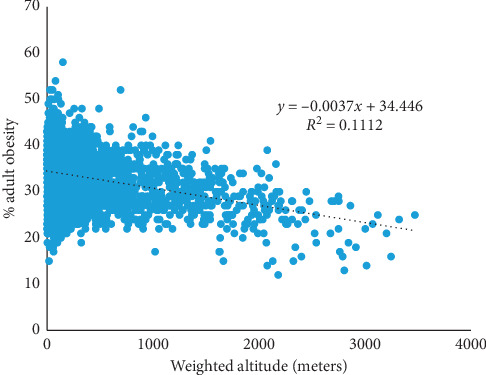
County-level adult obesity by altitude in the United States, 2016.

Prevalence (%) of adult obesity across the U. S. counties ranges from 12.0 to 58.0, with mean 32.9 (SD = 5.4) and median 33.0. A negative association exists between increasing weighted altitude and adult obesity (Figure 1). Eight states have average county-level weighted altitude at least 1,000 meters (Colorado, 2123.22; Utah, 1824.49; Wyoming, 1718.72; New Mexico, 1694.95; Nevada, 1545.51; Idaho, 1370.68, Montana, 1208.85, and Arizona, 1103.49). For these states, mean % adult obesity is 28.18 (SD = 5.28) compared with 33.34 (SD = 5.32) for the remaining states (*p* < 0.0001).

Summary statistics for altitude and other variables appear in Table 1. The table also shows the strength of the linear association between % adult obesity and the selected variables. The distribution of each variable tends to be normal, with exceptions for altitude and race. Most counties are below 500 meters (77.70%). The range for each variable varies considerably across counties. The strongest negative associations with adult obesity involve median household income, % some college, altitude, and Food Environment Index. The strongest positive associations with adult obesity involve % adult physically inactive, % adult smokers, % food insecure, % black, average PM2.5, average daily precipitation, and average daily maximum air temperature.

Associations between the altitude and the variables in this study are shown in Table 2. Adult obesity consistently decreases with increasing altitude; mean % adult obesity is 11.87 (or 35.28%) lower for counties ≥2,500 meters compared with <500 meters. Several other variables shown in the table are also associated with altitude. For example, the highest altitude group compared with the lowest altitude group has 6.05% higher average daily sunlight, 2.71% higher % non-Hispanic white, 116.69% higher % Hispanic, 36.26% higher % rural, 16.4% higher % some college, 16.13% higher median household income, 54.24% higher % with limited access to healthy foods, and 31.16% higher Food Inventory Index. They also have 30.46% lower average daily maximum air temperature, 55.54% lower average PM2.5, 49.51% lower average daily precipitation, 4.88% lower % female, 93.63% lower % black, 21.96% lower % food insecurity, 22.05% lower % adult smokers, and 36.78% lower % adult physical inactivity.

Adult obesity was regressed on the variables considered in this study, with their simultaneous relative contribution to the model identified (Table 3). Partial *R*^2^ measures the amount of variability in obesity associated with an independent variable when other variables are already included in the model. Variation in the percent of adult obesity across counties is primarily explained by adult physical inactivity (31.32%). After accounting for physical inactivity, smoking explained another 5.55% of the variation, and so on. Altitude in the absence of other variables in the model explains 11.12% of the variation in adult obesity. However, after accounting for adult physical inactivity and smoking, it explains 2.17% of the variation in adult obesity. Among the environmental variables, altitude has the greatest significant association with adult obesity followed by average daily maximum air temperature and average daily precipitation.

Mean % adult obesity in 2016 across counties in the contiguous U. S. is shown for the categories of weighted altitude in Table 4. As identified earlier, prior to adjustment, the mean % adult obesity is 35.28% lower in counties ≥2,500 meters compared with <500 meters. After adjusting for % adult physical inactivity, this value changes to 20.60% lower; after adjusting for % adult smokers, this value changes to 27.55% lower; and after adjusting for all the variables, this value changes to 17.54% lower.

## 4. Discussion

The nationally representative ecologic data confirmed the hypothesis of an inverse association between altitude and adult obesity after adjustment for several variables (see Tables 3 and 4). This result is consistent with other studies [8–10]. Variables that had the largest simultaneous relative effect on the association between altitude and adult obesity were adult physical inactivity and adult smoking. Each of these variables correlated with both altitude and adult obesity. Together, they explained 36.87% of the variation in adult obesity. Adult physical inactivity alone contributed to 31.32% of the variation in adult obesity.

Various mechanisms have been proposed in the literature for explaining the negative association between adult obesity and altitude. In a review study of the circulatory and metabolic responses to hypoxia in humans [18], the authors presented studies supporting hypoxia as a possible treatment for obesity [19, 20]. One study found that combining hypoxic exposure with exercise training may provide some additional health benefits, albeit limited, to standard normoxic exercise training for obese individuals [21]. In a review article involving obese individuals, there was little evidence that hypoxia had superior health effects (i.e., lower glucose, insulin, cholesterol, HDL, triglycerides, heart rate, blood pressure, body mass index, and body weight) compared with normoxia [22].

Hypoxia can occur at altitudes of 1,500 meters or higher [23]. Physiological responses increase with more severe hypoxia, which can occur from higher altitude exposure [18]. Hypoxia may change glucose metabolism and control appetite by altering the function of the nervous system and hormonal levels (e.g., plasma leptin) [19, 20, 24–27]. Hypoxia associated with higher altitude may also be protective against diabetes, cancer, heart disease, and stroke [28–32].

Previous results suggest a synergistic effect of physical activity and hypoxic exposure on body weight that may underlie the beneficial effect of living at altitude [33]. This is consistent with the finding in the current study that physical inactivity was not sufficient to explain obesity. It has been shown that the combination of hypoxic exposure and exercise compared with exercise alone produces more favorable improvements in fasting insulin, insulin sensitivity, triglycerides, and body fat content [34, 35]. Furthermore, as hypoxia contributes to better cardiovascular health and positive clinical implications [36, 37], greater physical activity may be possible.

Norepinephrine has also been suggested as a potential mechanism affecting the association between altitude and body weight [38]. Research has found that plasma norepinephrine concentrations significantly increase with increasing elevation [39]. The increase then suppresses blood flow to the intestines and, consequently, restricts appetite [40].

Although these mechanisms help explain why altitude continues to relate to adult obesity after adjustment for physical inactivity, tobacco smoking, and other variables, it is interesting to consider the influence of these other variables on the association between altitude and adult obesity. In support of our original hypothesis, physical inactivity and tobacco smoking appear to mediate some of the associations between altitude and adult obesity. The path of mediation may also include other variables. For example, physical inactivity decreases with higher altitude, most likely as a function of lower tobacco smoking, PM2.5, precipitation, air temperature, and other factors (1–5; Table 2). In multiple regression, the estimated association between altitude and obesity will appear less pronounced as mediators are entered into the model, as observed. Further research involving longitudinal data is required to establish temporality of events before statements about mediation can be conclusive.

Heavy smoking has been linked with greater body weight [41], as observed in the current study (see Tables 1 and 3). Some of the influences of smoking on obesity may be attributed to altitude. At higher altitude, smoking levels decrease. Since physical activity is more demanding (from a cardiorespiratory point of view) at higher altitude, individuals may feel the limiting effects of smoking on physical activity [42, 43] at any altitude greater than sea level, which may possibly increase their motivation to stop smoking.

After accounting for % adult physically active, % adult smokers, and altitude, which together explained 39.04% of the variation in adult obesity, all the remaining variables combined only contributed to 4.69% of the variation in adult obesity (see Table 3). However, some of these remaining variables (e.g., median household income, college education, Food Environment Index, PM2.5, precipitation, and air temperature) are still important on influencing obesity but primarily through their association with physical inactivity and smoking.

### 4.1. Limitations

The ecologic data used in the current study limit us to statistical model assessment of associations among variables. Statements about causality require more than just a valid statistical association but information about the temporality of events and other considerations such as biologic plausibility. Although we address some of the points in the literature on the mechanisms by which altitude may relate to body weight, further research involving longitudinal data is necessary to establish temporality. In addition, data related to physical inactivity were not available, such as sitting, television watching, and sedentary behavior. Research has shown that these variables play an independent role in terms of obesity, even after adjustment for several potential confounders, like predisposition for obesity [44]. Future research involving altitude and obesity may benefit by considering these variables. Finally, research shows increased metabolic expenditure required by the body to deal with very cold or hot temperatures [45]. However, determining the influence of outdoor air temperature on adult obesity in the current study was challenged because we have largely engineered ambient air temperature differences out of our lives through climate-controlled houses, workplaces, and vehicles.

## 5. Conclusions

Several variables attempted to explain the inverse association between altitude and adult obesity. However, higher altitude continued to be inversely associated with adult obesity after adjusting for these variables. Various mechanisms were discussed that help explain why higher altitude may lower the risk of adult obesity. In addition, higher altitude indirectly influenced adult obesity, primarily through its relationship with physical inactivity, but also through smoking. Adult obesity was most strongly associated with physical inactivity followed by adult smoking, altitude, average daily sunlight, and average daily precipitation.

## Figures and Tables

**Table 1:** Summary of selected county-level variables in the contiguous United States.

	No.	Mean	SD	Median	Min	Max	Pearson correlation coefficient with % adult obesity
*Environmental*							
Altitude (m)—weighted	3,106	414.3	487.4	263.1	−9.5	3471.4	−0.333
Altitude < 500 meters	2,412	208.2	129.2	212.3	−9.5	500.0	
Altitude 500−999 meters	367	699.7	140.8	676.3	500.6	997.0	
Altitude 1,000−1,499 meters	169	1,226.4	150.2	1,207.0	1,002.6	1,498.1	
Altitude 1,500−1,999 meters	88	1,721.5	150.6	1,699.6	1,500.5	1,993.7	
Altitude 2,000−2,499 meters	47	2,185.2	134.4	2,162.0	2,007.4	2,475.5	
Altitude 2,500 meters	23	2,885.1	257.9	2,804.8	2,524.6	3,471.4	
Avg daily sunlight (kJ/m^2^)	3,106	16,398.3	1,605.0	16,102.9	12,689.0	21,191.1	−0.024
Avg daily maximum air temperature (F)	3,106	65.4	9.3	64.8	38.4	87.5	0.227
Avg PM2.5 (*μ*g/m^3^)	3,108	9.0	2.0	9.4	3.0	19.7	0.260
Avg daily precipitation (mm)	3,106	2.7	0.9	3.0	0.2	7.1	0.236

*Demographic and lifestyle*							
% female	3,108	49.9	2.2	50.3	26.8	56.9	0.058
% non-Hispanic white	3,108	76.3	19.9	83.6	2.7	97.9	−0.089
% black	3,108	9.1	14.4	2.3	0.0	85.4	0.314
% Hispanic	3,108	9.7	13.9	4.4	0.6	96.4	−0.193
% rural	3,107	58.5	31.4	59.4	0.0	100.0	0.174
% some college	3,108	57.9	11.8	58.0	15.0	100.0	−0.372
Median household income	3,108	52,659.6	13,822.1	50,514.5	25,385.0	1,40,382.0	−0.427
% food insecure	3,108	13.28	3.97	13.00	3.00	36.00	0.367
% limited access to healthy foods	3,089	8.55	8.23	6.00	0.00	72.00	−0.001
Food Environment Index	3,089	7.5	1.1	7.7	0.0	10.0	−0.241
% adult smokers	3,108	17.4	3.6	17.0	6.0	41.0	0.501
% adult physical inactive	3,108	27.5	5.7	27.0	10.0	50.0	0.561

**Table 2:** Estimated mean and percent difference from the referent group for selected variables by altitude.

Variable	Altitude <500 meters	Altitude 500–999 meters	Altitude 1,000–1,499 meters	Altitude 1,500–1,999 meters	Altitude 2,000–2,499 meters	Altitude ≥2,500 meters
	*N* = 2412	*N* = 367	*N* = 169	*N* = 88	*N* = 47	*N* = 23
% adult obese	Referent	−1.74	−3.26	−5.64	−8.12	−11.87
	Referent	−5.18%	−9.69%	−16.77%	−24.14%	−35.28%
	A	B	B	C	D	E
Avg daily sunlight (kJ/m^2^)	Referent	148.12	964.99	982.50	1454.25	984.90
	Referent	0.91%	5.93%	6.04%	8.94%	6.05%
	A	A	B	B	B	B
Average daily max air temperature (F)	Referent	−2.40	−3.75	−9.93	−13.08	−20.25
	Referent	−3.61%	−5.64%	−14.93%	−19.66%	−30.46%
	A	A	B	C	D	E
Average PM2.5 (*μ*g/m^3^)	Referent	−2.28	−3.17	−3.58	−4.47	−5.37
	Referent	−23.60%	−32.78%	−37.04%	−46.19%	−55.54%
	A	B	C	C	D	E
Average daily precipitation (mm)	Referent	−1.04	−1.71	−1.87	−1.79	−1.50
	Referent	−34.16%	−56.44%	−61.85%	−58.90%	−49.51%
	A	B	C, D	D	D	C
% female	Referent	−0.84	−0.96	−1.52	−1.41	−2.45
	Referent	−1.68%	−1.91%	−3.04%	−2.82%	−4.88%
	A	B	B	B	B	C
% non-Hispanic white	Referent	3.25	−5.31	−0.12	−6.64	2.07
	Referent	4.26%	−6.96%	−0.16%	−8.70%	2.71%
	A	A	B	A	B	A
% black	Referent	−9.52	−9.89	−9.98	−10.50	−10.54
	Referent	−84.59%	−87.91%	−88.66%	−93.31%	−93.63%
	A	B	B	B	B	B
% Hispanic	Referent	4.66	13.98	7.67	13.20	9.21
	Referent	59.03%	177.09%	97.15%	167.16%	116.69%
	A	B	C	B	C	B, C
% rural	Referent	16.30	3.95	-0.19	5.94	20.36
	Referent	29.03%	7.03%	-0.34%	10.58%	36.26%
	A	B	A	A	A	B
% some college	Referent	9.43	8.15	9.21	5.59	5.06
	Referent	2.23%	0.38%	6.67%	7.59%	16.40%
	A	B	B	B	B	B
Median household income	Referent	−3019.97	−2993.77	4586.60	4366.45	8536.84
	Referent	−5.71%	−5.66%	8.67%	8.25%	16.13%
	A	A, B	A, B	A, C, D	A, C, D	D
% food insecure	Referent	−1.64	−1.10	−0.99	−0.55	−2.99
	Referent	−12.07%	−8.12%	−7.30%	−4.06%	−21.96%
	A	A	A	A	A	B
% limited access to healthy foods	Referent	5.78	6.34	5.33	6.54	3.93
	Referent	79.83%	87.45%	73.64%	90.31%	54.24%
	A	B	B	B	B	B
Food Environment Index	Referent	−0.20	−0.35	−0.30	−0.49	0.24
	Referent	−2.63%	−4.66%	−3.93%	−6.51%	3.16%
	A, B	A, B	A	A	A	B
% adult smokers	Referent	−1.37	−2.27	−3.21	−3.69	−3.95
	Referent	−7.65%	−12.65%	−17.93%	−20.62%	−22.05%
	A	B	B, C	C, D	D	D
% adult physical inactive	Referent	−0.83	−2.77	−5.41	−7.08	−10.32
	Referent	−2.97%	−9.87%	−19.29%	−25.24%	−36.78%
	A	A	B	C	C	D

*Note.* Mean percent difference scores are compared among groups using Student–Newman–Keuls' (SNK) multiple-range test. Corresponding to the SNK test are capital letters, which identify whether significant differences exist in the estimates across the levels of altitude. There is a significant difference in means between groups if the letters differ. For example, with % adult obesity, the mean percent difference scores between the first and second altitude groups are significant, but there is no difference in mean percent difference scores between the second and third altitude groups.

**Table 3:** Contributions of selected variables to the variation in % adult obesity in 2016 across counties in the United States.

Variable	Parameter estimate	Standard error	*F* value	Pr > *F*	Partial *R*^2^ (%)	Model *R*^2^ (%)	*F* value	Pr > *F*
% adult physically inactive	0.32	0.02	306.71	<0.0001	31.32	31.32	1405.94	<0.0001
% adult smokers	0.25	0.04	46.66	<0.0001	5.55	36.87	271.07	<0.0001
Altitude-weighted	−0.0018	0.0003	40.41	<0.0001	2.17	39.04	109.71	<0.0001
Average daily sunlight (kJ/m^2^)	−0.0007	0.0001	30.67	<0.0001	1.60	42.46	85.32	<0.0001
Average daily precipitation (mm)	−1.13	0.13	76.09	<0.0001	1.02	40.07	52.62	<0.0001
% black	0.04	0.01	10.32	0.0013	0.79	40.86	41.31	<0.0001
% some college	−0.04	0.01	17.16	<0.0001	0.57	43.03	30.78	<0.0001
Median household income	−0.00005	0.00001	23.15	<0.0001	0.23	43.25	12.45	0.0004
Food Environment Index	−0.36	1.13	0.10	0.748	0.17	43.42	9.11	0.0026
% non-Hispanic white	−0.03	0.01	6.87	0.0088	0.10	43.67	5.58	0.0182
% female	0.06	0.04	3.01	0.0831	0.08	43.50	4.20	0.0405
Average daily max air temperature (F)	0.04	0.02	2.91	0.088	0.03	43.53	1.69	0.1931
% Hispanic	−0.05	0.02	9.03	0.0027	0.03	43.56	1.85	0.1743
% food insecure	−0.17	0.21	0.68	0.4107	0.03	43.72	1.50	0.2209
Average daily PM2.5	0.07	0.06	1.58	0.2089	0.02	43.69	1.31	0.2516
% limited access to healthy foods	−0.06	0.10	0.33	0.5665	0.01	43.72	0.33	0.5658
% rural	−0.001	0.003	0.06	0.8093	0.00	43.73	0.06	0.8093

*Note. R* ^2^ is a measure of the proportion of the variance for a dependent variable that is explained by an independent variable or variables within a regression model. Partial *R*^2^ measures the marginal contribution of a given independent variable when another variable is already included in the model. Model *R*^2^ is a measure of the cumulative partial *R*^2^ values, increasing with each additional variable added to the model.

**Table 4:** Adjusted estimates of % adult obese in 2016 according to categories of altitude, counties in the contiguous U.S.

			Adjusted for					
			% adult physical inactivity		% adult smokers		All variables^*∗*^	
	Mean (%)		Mean (%)		Mean (%)		Mean (%)	
<500 meters	33.66	Referent	33.37	Referent	33.34	Referent	33.35	Referent
500–999 meters	31.91	−5.18%	32.03	−4.01%	32.53	−2.43%	32.06	−3.87%
1,000–1,499 meters	30.40	−9.69%	31.45	−5.75%	31.62	−5.15%	31.47	−5.64%
1,500–1,999 meters	28.01	−16.77%	30.35	−9.06%	29.88	−10.38%	30.67	−8.03%
2,000–2,499 meters	25.53	−24.14%	28.68	−14.07%	27.73	−16.83%	29.55	−11.41%
2,500 meters	21.78	−35.28%	26.50	−20.60%	24.16	−27.55%	27.50	−17.54%
*F* value	78.56		34.03		42.00		11.09	
Pr > *F*	<0.0001		<0.0001		<0.0001		<0.0001	

^*∗*^Average maximum air temperature and precipitation and average fine particulate matter, % female, % non-Hispanic white, % black, % Hispanic, % rural, % some college, median household income, % food insecure, Food Environment Index, % smokers, and % physically inactive.

## Data Availability

The data are in the public domain and accessible through the references provided in the paper. Data are also available from the author.

## Additional Points

*Already known.* Previous studies have identified an inverse association between altitude and obesity after adjusting for selected variables, including physical activity. Possible mechanistic explanations have been presented to explain the inverse association. *What this study adds*. Statistical description of relationships between several variables and altitude and between these same variables and adult obesity. The contribution of these variables to the inverse association between altitude and adult obesity is presented and discussed. *Future research and value to clinical practice*. This study identified areas for future research: (1) using a longitudinal design to better identify the temporal sequence of events, (2) considering other measures of physical inactivity, including sitting, television viewing, and sedentary behavior, as possible intermediate factors in the relationship between altitude and obesity, and (3) assessing the association between altitude and pediatric obesity. The paper identifies vulnerable populations and provides information that may be helpful in developing obesity prevention strategies.
